# The Week's Work

**Published:** 1910-10-01

**Authors:** 


### THE WEEK'S WORK.

### LONGFORD INFIRMARY AND ITS SURGEON.

The Dublin papers contain the report of a recent meeting of the governing body of Longford County Infirmary, a small but very old hospital in Ireland, which has just dismissed its surgeon, the medical officer of the infirmary, who is, though an old man, still active and energetic. Owing to some friction between him and one of the nurses the committee of the infirmary in July last called upon him to resign. This he has refused to do, protesting that any want of harmony in the institution has not been caused through his action. At the last meeting of the committee it was made clear that the governing body was quite willing to grant Dr. Mayne a gratuity or retiring allowance. In view of the fact that he had refused to resign some feeling appeared to have been aroused, and a motion was tabled recommending his instant dismissal. After discussion the motion was amended so as to provide for the doctor's dismissal subject to the approval of the Local Government Board after inquiry into the whole matter, with an added proviso that in case Dr. Mayne tendered his resignation before the matter came before the Local Government Board, it be accepted, and the maximum retiring allowance made to him. At the same meeting the resignation of the nurse who had complained of want of harmony between herself and the medical officer in carrying on the work of the institution was read and accepted. Dr. Mayne qualified in 1871, and has been associated with the infirmary for the past thirty years. The action of the committee, we understand, has been largely prompted by the feeling that he is too old for the post, and it is only fair that he should accept the conclusion arrived at, however unpalatable it may be, since it casts no reflection whatever upon his administrative and professional competency. It is perfectly fair for a hospital board to lay down an age limit, and although it appears that the discussion in this case was not wholly free from personalities, the committee seems to have acted in a fair and gentlemanly manner in first approaching the surgeon before taking action to secure his retirement.

### THE POOR-LAW COMMISSION'S REPORT.

The *Sheffield Weekly News* has reprinted, in pamphlet form, the series of nine lectures delivered on the report of the Royal Commission on the Poor Laws and the Relief of Distress, by the Dean of Norwich, Mr. Tawney, Mrs. Sidney Webb, the Rev. L. R. Phelps, Mr. Manton, Mr. Bentham, Dr. MacVail, Dr. Bosanquet, and Miss Phillips. The pamphlet, which is issued at the modest price of fourpence, is prefaced by a brief introduction from the pen of the local medical officer of health, who very impartially sets out the findings of the commissioners and leaves his readers to draw their own conclusions from the evidence. The lectures differ much in subject and quality, though they all have this in common that they plead largely for the adoption of either the one or other of the reports. Those delivered by Mrs. Webb and Dean Wakefield, for example, are frankly bits of special pleading, which do not materially contribute to the understanding of the Minority Report. Mr. Tawney's lecture on "Boy Labour," however, is an excellent summary of the case, and considerably elucidates both reports, though Mr. Tawney himself supports the Minority proposals. Dr. Bosanquet's essay is a very readable one, with which most thoughtful readers will agree in the main. He lays stress on the outspoken hostility to voluntary charity, which is one of the features of the Minority Report. The question of provident treatment and State insurance is dealt with, though hardly in so thorough and illuminative a manner as we should like to see it treated in a popular pamphlet. On the whole, the little booklet is a valuable contribution to the controversy, and its simplicity makes it attractive to the busy man who has little time to wade through longer and more pretentious articles.

### THE NEWCASTLE INFIRMARY CHAPEL DISPUTE.

This wearisome dispute, after a settlement had, it was fairly confidently expected, been at last arrived at, nevertheless is now protracted a stage further. It will be remembered that the quarterly Court of Governors issued instructions to the House Committee to open the chapel and provide equal facilities for Anglican and Free Church worship there. This the House Committee has refused to do, its reason being that such an act might personally entail sundry legal penalties. This question of the consequences of a breach of ecclesiastical law had not been lost sight of previously. It was mentioned in the discussion of the whole matter by the Governors, and the general feeling was that it was extremely unlikely anyone would fly in the face of public opinion by resorting to any such litigation. This opinion we ventured to endorse, and it is again affirmed, we notice, both directly and by implication in the local Press. By their last action, then, members of the House Committee, who are credited with being originally responsible for the consecration of the chapel after the rites of the Church of England, have laid themselves somewhat open to the suspicion of being influenced by the famous *odium theologicum.* An example of liberality of sentiment was certainly set by the gentleman, who (as we noted in The Hospital of the 10th ult.), although evidently of anti-Christian belief, yet seconded a resolution in favour of reopening the chapel and thus rescinding a former resolution he had helped to pass. The contrast between Mr. Cairns's action and that of the House Committee is interesting to the student of human nature. However, we have no desire to be unsympathetic, and can only wish the Newcastle Infirmary authorities a happy deliverance from the recurrent waste of time these disputes mean.

### THE JEWISH MEMORIAL HOSPITAL.

On Sunday last a public meeting was held in support of the scheme to erect a special Jewish hospital as a memorial to the late King. Dr. A. Gaster, the honorary treasurer of the London Jewish Hospital Association, stated that a site had been bought for the erection of the proposed hospital. This site has cost £5,000, of which £3,400 is still unpaid. The hospital scheme was outlined, from which it appears that the committee proposes the erection of a hospital to consist of fifty beds and an out-patient department, at a cost of between £15,000 and £20,000—a sum which seems to us a very low estimate indeed. A point is made of the fact that the hospital will not be for the exclusive use of persons of the Jewish faith, but will be open to the sick of all creeds. The meeting finally passed a resolution affirming its determination to proceed with the establishment of the proposed institution. It is, we believe, three years since the proposal was originally mooted that the Jews in London should erect a hospital of their own. At that time the facilities granted to persons of the Jewish faith in several London hospitals were meagre in the extreme; since then, however, they have been greatly modified and improved, and when one considers the excellent Jewish wards at the London, and the strong support given by the Jews to the German and Metropolitan hospitals, it would seem that the objections which were then raised are no longer valid. In the circumstances we fail to see the advantage, to the Jews or to the community at large, of building a special hospital, which, to judge from the outlined scheme, must be a small one. In view of the comparatively slight support that the whole scheme has hitherto received, we sincerely trust that the committee will not proceed with the scheme, but bring forward a proposal to use the funds already subscribed for the establishment either of special Jewish wards in the East-End hospitals, or, if it is absolutely necessary that the purchased site must be retained for sentimental reasons, to establish an up-to-date Jewish dispensary. There is no doubt that, on the whole, the Jews are satisfied with the present hospital arrangements, and there is certainly not the same reason for establishing a special hospital as existed at Berlin at the time when the Jewish Hospital there was founded. The whole plan outlined at last Sunday's meeting appears to us to be open to grave objection, and its execution will serve no useful purpose, at least at the present moment, but rather, on the contrary, increase the difficulties of those East-End institutions which have hitherto done their best to grant all possible facilities to Jew patients.

### THIS WEEK'S DRUG MARKET.

The improved tone of the drug market which has been noticeable during the last few weeks continues. On the whole prices are well maintained, but in the case of two or three articles there is a tendency to lower prices. Cocaine hydrochloride is 4d. per oz. dearer, but the present value is very low compared with the prices which were formerly quoted for this drug. Seidlitz powder and tartrated soda are both rather dearer. Cream of tartar and tartaric acid still show an advancing tendency. Menthol and peppermint oil are again dearer. Aniseed oil is also slightly dearer. On the other hand, opium is very slightly lower in price, but it is impossible to form any reliable opinion as to whether the slight falling-off in value is only of a temporary character. Morphine and codeine are unchanged in price. Buchu leaves are again lower in value. East Indian ipecacuanha is much cheaper, but the price of Rio ipecacuanha is fairly well maintained. A further advance in the price of glycerine is expected.

